# uSing rolE-substitutioN In care homes to improve ORal health (SENIOR): a study protocol

**DOI:** 10.1186/s13063-022-06487-3

**Published:** 2022-08-18

**Authors:** Gerald McKenna, Georgios Tsakos, Sinead Watson, Alison Jenkins, Patricia Masterson Algar, Rachel Evans, Sarah R. Baker, Ivor G. Chestnutt, Craig J. Smith, Ciaran O’Neill, Zoe Hoare, Lynne Williams, Vicki Jones, Michael Donaldson, Anup Karki, Caroline Lappin, Kirstie Moons, Fiona Sandom, Mary Wimbury, Lorraine Morgan, Karen Shepherd, Paul Brocklehurst

**Affiliations:** 1 Centre for Public Health, School of Medicine, Dentistry and Biomedical Sciences, Queen’s University Belfast, Institute of Clinical Science Block A, Belfast, BT12 6BA UK; 2 Department of Epidemiology and Public Health, University College London, London, UK; 3 NWORTH Clinical Trials Unit, Bangor University, Bangor, UK; 4 School of Health Sciences, Bangor University, Bangor, UK; 5 Unit of Oral Health, Dentistry and Society, University of Sheffield, Sheffield, UK; 6 College of Biomedical and Life Sciences, Cardiff University, Cardiff, UK; 7 Division of Cardiovascular Sciences, Lydia Becker Institute of Immunology and Inflammation, University of Manchester, Manchester, UK; 8 Manchester Centre for Clinical Neurosciences, Manchester Academic Health Science Centre, Geoffrey Jefferson Brain Research Centre, Salford Royal Foundation NHS Trust, Salford, UK; 9 Community Dental Services, Aneurin Bevan University Health Board, Newport, UK; 10 Health and Social Care Board, Belfast, UK; 11 Public Health Wales, Cardiff, UK; 12 Community Dental Service, South Eastern Health and Social Care Trust, Dundonald, UK; 13 Health Education and Improvement Wales, Nantgarw, UK; 14 Care Forum Wales, Wrexham, UK; 15 Patient and Public Involvement, Newport, UK; 16 Patient and Public Involvement, Chester, UK

**Keywords:** Cluster-randomised controlled trial, Oral health, Dental Care Professionals, Intervention, Care homes, Older adults

## Abstract

**Background:**

Dental service provision in the care home sector is poor, with little emphasis on prevention. Emerging evidence suggests that the use of Dental Care Professionals (dental therapists and dental nurses) as an alternative to dentists has the potential to improve preventive advice, the provision of care and access to services within care homes. However, robust empirical evidence from definitive trials on how to successfully implement and sustain these interventions within care homes is currently lacking. The aim of the study is to determine whether Dental Care Professionals could reduce plaque levels of dentate older adults (65 + years) residing in care homes.

**Methods:**

This protocol describes a two-arm cluster-randomised controlled trial that will be undertaken in care homes across Wales, Northern Ireland and England. In the intervention arm, the dental therapists will visit the care homes every 6 months to assess and then treat eligible residents, where necessary. All treatment will be conducted within their Scope of Practice. Dental nurses will visit the care homes every month for the first 3 months and then three-monthly afterwards to promulgate advice to improve the day-to-day prevention offered to residents by carers. The control arm will be ‘treatment as usual’.

Eligible care homes (*n* = 40) will be randomised based on a 1:1 ratio (20 intervention and 20 control), with an average of seven residents recruited in each home resulting in an estimated sample of 280. Assessments will be undertaken at baseline, 6 months and 12 months and will include a dental examination and quality of life questionnaires. Care home staff will collect weekly information on the residents’ oral health (e.g. episodes of pain and unscheduled care). The primary outcome will be a binary classification of the mean reduction in Silness-Löe Plaque Index at 6 months. A parallel process evaluation will be undertaken to explore the intervention’s acceptability and how it could be embedded in standard practice (described in a separate paper), whilst a cost-effectiveness analysis will examine the potential long-term costs and benefits of the intervention.

**Discussion:**

This trial will provide evidence on how to successfully implement and sustain a Dental Care Professional-led intervention within care homes to promote access and prevention.

**Trial registration:**

ISRCTN16332897. Registered on 3 December 2021.

**Supplementary Information:**

The online version contains supplementary material available at 10.1186/s13063-022-06487-3.

## Administrative information

Note: the numbers in curly brackets in this protocol refer to SPIRIT checklist item numbers. The order of the items has been modified to group similar items (see https://www.equator-network.org/reporting-guidelines/spirit-2013-statement-defining-standard-protocol-items-for-clinical-trials/).

**Table:** 

Title {1}	**uSing rolE-substitutioN In care homes to improve ORal health (SENIOR): A study protocol**
Trial registration {2a and 2b}.	ISRCTN16332897
Protocol version {3}	Version 3: 18/03/2021
Funding {4}	This study is supported by a grant (NIHR128773) from the National Institute for Health Research awarded in 2018 (under the Health Services and Delivery Research Programme).
Author details {5a}	Gerald McKenna^1^, Georgios Tsakos^2^, Sinead Watson^1^, Alison Jenkins^3^, Patricia Masterson Algar^4^, Rachel Evans^3^, Sarah R Baker^5^, Ivor G Chestnutt^6^, Craig Smith^7,8^, Ciaran O’Neill^1^, Zoe Hoare^3^, Lynne Williams^4^, Vicki Jones^9^, Michael Donaldson^10^, Anup Karki^11^, Caroline Lappin^12^, Kirstie Moons^13^, Fiona Sandom^13^, Mary Wimbury^14^, Lorraine Morgan^15^, Karen Shepherd^15^ and Paul Brocklehurst^3^. ^*1*^*Centre for Public Health, School of Medicine, Dentistry and Biomedical Sciences, Queen’s University Belfast, Belfast, UK; *^*2*^*Department of Epidemiology and Public Health, University College London, London, UK; *^*3*^*NWORTH Clinical Trials Unit, Bangor University, Bangor, UK; *^*4*^*School of Health Sciences, Bangor University, Bangor, UK; *^*5*^*Unit of Oral Health, Dentistry and Society, University of Sheffield, Sheffield, UK; *^*6*^*College of Biomedical and Life Sciences, Cardiff University, Cardiff, UK; *^*7*^*Division of Cardiovascular Sciences, Lydia Becker Institute of Immunology and Inflammation, University of Manchester, Manchester, UK; *^*8*^*Manchester Centre for Clinical Neurosciences, Manchester Academic Health Science Centre, Geoffrey Jefferson Brain Research Centre, Salford Royal Foundation NHS Trust, Salford, UK; *^*9*^*Community Dental Services, Aneurin Bevan University Health Board, UK; *^*10*^*Health and Social Care Board, Belfast, UK; *^*11*^*Public Health Wales, Cardiff, UK; *^*12*^*Community Dental Service, South Eastern Health and Social Care Trust, Dundonald, UK; *^*13*^*Health Education and Improvement Wales, UK; *^*14*^*Care Forum Wales, UK; *^*15*^*Patient and Public Involvement.*
Name and contact information for the trial sponsor {5b}	Dr Huw Roberts, College of Human Sciences *Bangor University* *Gwynedd* *LL57 2DG* *Tel: 01248 383136* *Email:*huw.roberts@bangor.ac.uk
Role of sponsor {5c}	The sponsor has ultimate responsibility for the initiation, management (or arranging the initiation and management) and/or financing (or arranging the financing) for the SENIOR study. The sponsor takes primary responsibility that the design of the SENIOR study meets appropriate standards and that arrangements are in place to ensure appropriate conduct and reporting.

## Introduction

### Background and rationale {6a}

Poor oral health, including dental caries and periodontal disease, is a very common problem for older adults residing in care homes and the issue is increasingly becoming a significant public health problem [1, 2]. Amongst older adults, 40% of the 75–84 age group and 33% of the 85 + age group have dental caries, whilst periodontal disease affects 69% of those over 65 years of age [3]. The oral health of care home residents is much worse than their community living peers. With increasing dependency, the ability for self-care deteriorates, poly-pharmacy leads to dry mouth and diets become rich in sugars [4]. All these factors significantly increase residents’ disease burden and the risk of future problems. Oral conditions impact on their quality of life, self-esteem, general health and diet, exacerbating underlying medical conditions [4–7]. Income-related inequality in oral health of older adults is also a major issue [8, 9].

Despite this high level of need, dental service provision in residential care is poor, with little emphasis on prevention [10, 11]. Access to domiciliary services is difficult and unscheduled care for dental problems (including hospital admissions) is common, complex to deliver and expensive [12, 13]. The World Health Organization (WHO) argue that the design of long-term care systems that are fit for ageing populations should take priority [14] and the Royal College of Surgeons of England (RCS) [15], Public Health England (PHE) [11] and the National Institute of Clinical Excellence (NICE NG48) [16] have all called for more high-quality research. Recently, the Care Quality Commission (CQC) have also highlighted the paucity of dental care in care homes in their Oral Health Care in Care Homes report published in June 2019 [17].

There is increasing evidence that Dental Care Professionals (DCPs) offer an alternative to using dentists to meet the future challenges in dental public health [18, 19]. DCPs are a broad range of professionals, which include dental therapists (DTs) and dental nurses (DNs). Previous research has demonstrated that DTs can identify and screen for dental caries and periodontal disease and are safe as front-line health care workers [20–22]. The feasibility, productivity and effectiveness of using DTs has been tested in primary care [21, 23, 24], but little has been undertaken in a care home environment. Emerging evidence suggests that the use of DCPs (DTs and DNs) within care homes has the potential to improve preventive advice, the provision of care and access to services [25].

Following a retrospective analysis of the 2010 Welsh dental care home survey data, Monaghan & Morgan [26] concluded ‘a large proportion of need in care homes could be wholly provided by hygienists or therapists’ and an ‘efficiency gain of direct access arises from individuals who do not need to see a dentist for any aspects of their care’. It is also argued that DTs can lead examinations in care home settings [27]. However, robust empirical evidence from definitive trials about how best to successfully implement and sustain DCP interventions within care home environments is currently lacking.

The aim of this study is to determine whether DCPs could reduce plaque levels (improve the oral cleanliness) of dentate older adults (over 65 years of age) residing in care homes over a 6-month period, when compared to ‘treatment as usual’ (commonly a reactive and ad hoc service provided by dentists), and also to determine whether this effect is sustainable over a further 6-month follow-up period.

### Objectives {7}

The objectives of the study are to:

1. Undertake a cluster-randomised controlled trial (RCT) across Wales, Northern Ireland and England to determine whether role-substitution using DCPs could reduce plaque levels (improve oral cleanliness) of dentate older adults residing in care homes over a 6-month period, when compared to ‘treatment as usual’;
2. Follow residents for a further 6 months to determine whether this effect is sustainable;
3. Use semi-structured interviews to undertake a process evaluation of the trial to determine acceptability, treatment fidelity and pathways-to-impact with the following stakeholders (detailed in a parallel paper):
  - Managers and staff of care homes to assess the intervention’s feasibility and sustainability;
  - Residents, relatives and informal carers to explore the intervention’s acceptability;
  - Managers and residents that refused participation to explore their narrative; and
  - Commissioners of care renew care-pathways and pathways-to-impact; and
4. Undertake a parallel cost-effectiveness analysis from a National Health Service (NHS) perspective.

### Trial design {8}

**Fig. 1:**
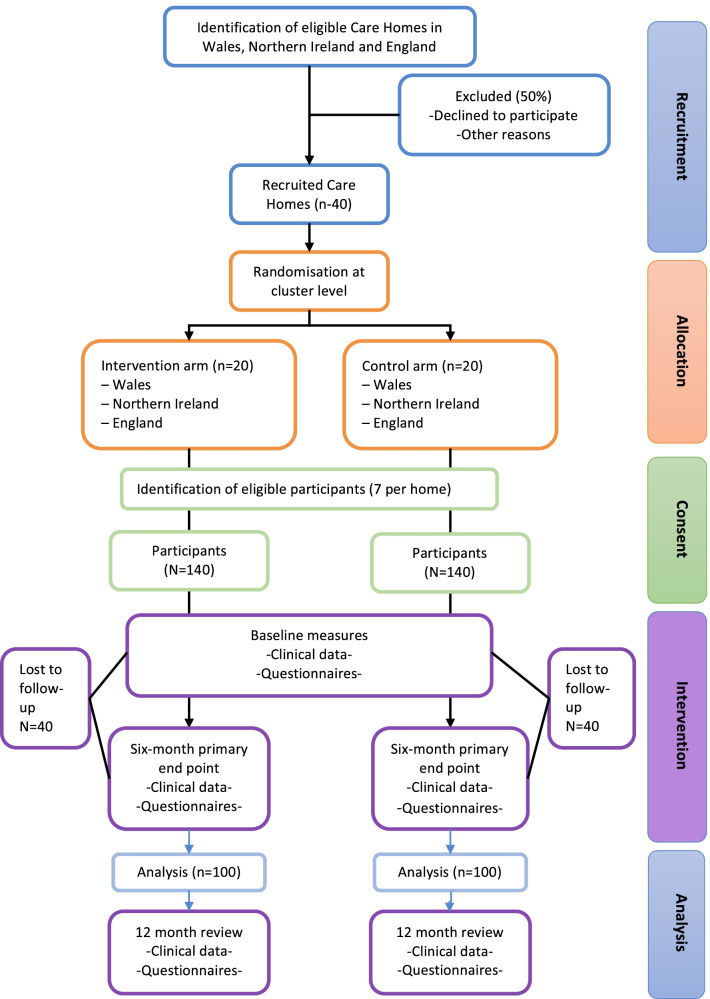
SENIOR trial flowchart

This is a multi-centre, two-armed, cluster-randomised trial, based on a superiority design, to determine whether DCPs could reduce levels of dental plaque of dentate older adults residing in care homes over a 6-month period, when compared with ‘treatment as usual’. Residents will be followed for a further 6 months to determine whether this effect is sustainable. Care homes (*n* = 40) will be randomised based on a 1:1 ratio (20 intervention and 20 control), with an average of seven residents recruited in each care home resulting in an estimated sample size of 280. Figure 1 provides an overview of the RCT’s design.

A cost-effectiveness analysis from a NHS perspective will examine the potential long-term costs and benefits of the intervention.

A process evaluation involving semi-structured interviews with key stakeholders will be undertaken alongside the trial to explore the intervention’s acceptability and treatment fidelity (subject of an additional paper). It will also capture the contextual factors that shape the intervention, mechanisms that sustain or potentiate effects, unexpected pathways and consequences and the contextual factors that shape implementation. A semi-structured interview schedule will be developed in collaboration with the patient and public involvement (PPI) group. Audio-taped interviews will be conducted both in person and using virtual technology. Data will be anonymised, fully transcribed and analysed by the researchers using thematic analysis.

## Methods: participants, interventions and outcomes

### Study setting {9}

The trial will be conducted in 40 care homes from across Wales, Northern Ireland and England (London and North England). Further information about recruitment and randomisation of care homes is presented in the relevant sections below. DTs and DNs employed by the Community Dental Service (CDS) in each region will deliver the oral health intervention. CDSs are charged with the responsibility of providing NHS service provision for care homes on a regional basis.

### Eligibility criteria {10}

#### Selection of care homes

##### Inclusion criteria

1. Minimum of ten residents aged 65 years and over.

##### Exclusion criteria

(2) Current participation in TOPIC (Improving the oral health of older people in care homes) [28], Gwên am Byth [29] or another oral health programme; and
(3) Care homes that only specialise in end−of−life or palliative care.

#### Selection of residents

Care home residents will be eligible to participate if they are able to meet the following inclusion criteria and have the capacity to provide consent:

1. 65 years and over;
2. Dentate or partially dentate (at least six natural teeth); and
3. Full−time resident in care facility.
  Exclusion criteria:
4. Residents who are only receiving end−of−life or palliative care.

### Who will take informed consent? {26a}

The local study team at each study site will obtain written informed consent from eligible care homes and eligible residents. Care homes and their residents interested in taking part in the RCT will be given a Participant Information Sheet (PIS) and an Informed consent Form (ICF). Written informed consent will be taken at least 48 h after informing the care homes and their residents about the study. As the project involves sites in Wales, to comply with the Welsh Language Act 1993, the relevant PISs and ICFs will be translated into Welsh and offered bilingually.

On entry into the study, the local study team will complete a Six-item Cognitive Impairment Test (6-CIT) [30] with residents in order to assess their level of cognitive function. Residents will be asked to consent for the 6-CIT screen and for the results to be fed back to the care home manager to discharge our duty of care.

Consent into the study will follow guidance laid down within the Mental Capacity Act 2005. Potential participants will be assumed as having capacity to consent for themselves unless formally assessed as lacking capacity, in which case the views of a Personal Consultee (usually a relative) will be sought. If no Personal Consultee can be identified, a Professional Consultee who is (a) independent of the study and (b) knows the participant well enough to consider their views will be approached. If the participant’s lack of capacity is considered temporary, capacity will be reassessed prior to each contact with the researcher. If a participant communicates his/her objection to a research assessment or intervention, either verbally or non-verbally, the intervention will cease immediately. Further attempts will be made at a later time; however, if the participant continues to object then he/she will be withdrawn from the study. The safety and well-being of residents will be paramount at all times.

### Additional consent provisions for collection and use of participant data and biological specimens {26b}

Not applicable as no biological specimens will be collected as part of this trial.

## Interventions

### Explanation for the choice of comparators {6b}

The control will be routine practice. The results from a PHE survey [10] and a Priority Setting Partnership exercise undertaken with four stakeholder groups, including service users, carers, third sector organisations and oral health specialists [31], suggest that this practice is likely to be heterogeneous and ad hoc, including intermittent domiciliary care and infrequent tooth-brushing with toothpaste by unsupervised residents.

### Intervention description {11a}

The description of the intervention below has been reported in accordance with the TIDieR (Template for Intervention Description and Replication) guidelines [32].

DTs will visit care homes in their locality every 6 months, and the DNs will visit every month for the first 3 months and then 3-monthly afterwards. This approach builds on the existing Gwên am Byth programme (which has not been empirically evaluated) [29]. In this manner, DTs will proactively oversee the clinical management of eligible dentate residents. The DTs will provide any simple operative treatment for individual residents, within their Scope of Practice (referring onto dentists within the CDS when necessary) [33]. Monaghan and Morgan [26] found that these management strategies would address the majority of cases that are likely to be seen in residents based in care homes. Extractions will need to be referred to dentists and DTs will act to sign-post residents to the CDS. Other clinical cases which may require onwards referral are fractured dentures, crowns and bridges, but these are less likely to warrant further intervention, given the focus on palliative management in this context [34].

The DNs will promulgate advice to improve the day-to-day prevention offered to residents based on Delivering Better Oral Health [35]. This will include the following:

- Professional application of prescribed fluoride (2.2% NaF varnish) every 3 months
- 5000 ppm fluoride toothpaste, prescribed as appropriate
- Oral hygiene advice; and
- Recommendation of the Eatwell Guide [36].

The visits from the DNs will form an important function in terms of championing oral health amongst care home managers and staff. This element of the complex intervention is just as important as the 6-monthly clinical management of dental need by the DTs. As highlighted by Brocklehurst et al. [37], ‘there is growing support for the use of change agents in implementation processes’. Human agency where clinical or non-clinical staff act as change agents to facilitate the enactment of complex interventions is increasingly seen to be key [38–40]. To facilitate this process, training materials will be provided using the All-Wales Faculty of Dental Care Professionals platform [41]. This will include dedicated documents for care home staff, based on a funded study that has utilised co-production and co-design principles to adapt NICE guidelines NG48 (TOPIC) [28]. Hard-copy training manuals will also be provided to care homes.

The local study team will be responsible for reviewing the delivery of the intervention so that it meets the criteria of the trial, i.e. a visit by the DT at least once every 6 months and by the DN once a month in the first quarter and then 3-monthly afterwards.

### Criteria for discontinuing or modifying allocated interventions {11b}

Care homes and residents will be able to withdraw at any time during the study. Where possible, in agreement with participants, data from those withdrawn will be used in analysis unless consent for this is specifically withdrawn.

### Strategies to improve adherence to interventions {11c}

There will be no additional strategies to improve the adherence to the intervention. Adherence to the intervention will be monitored by collecting information on completion rates (fully, partially or not completed) for the following: (1) weekly oral symptoms checklist collected by care home staff and (2) completed care home staff training.

### Relevant concomitant care permitted or prohibited during the trial {11d}

The intervention will be delivered alongside any oral care practices currently in place in the care homes. Care homes will not be asked to cease any practices that they are currently undertaking in either the control or intervention arm.

### Provisions for post-trial care {30}

Cover for harm as a result of the design or conduct of the trial has been arranged with the study Sponsor (Bangor University). In the event of complaints and concerns, these can be directed towards the research team, and participants will have the relevant contact details. Clinical treatment provided by the DTs and DNs will be covered by their individual or organisation’s indemnity. Care homes in the intervention arm can retain the hard-copy training manuals. The training manuals will also be made available to the control arm care homes after the end of the study.

### Outcomes {12}

#### Primary outcome in the trial

The Primary Outcome Measure (POM) for this study will be the Silness-Loë Plaque Index [42]. The index is a 4-point scale (measuring the amount of dental plaque present on six index teeth) ranging from 0-no plaque to 3-abundance of dental plaque within the gingival pocket and/or on the tooth and gingival margin. In accordance with the index, the mean will be calculated from these ordinal scores on the six index teeth. The prevailing model in the dental literature is to measure the change in this mean score. However, this model is difficult to infer clinical significance at a population level. To this end, the proportion of individuals in each arm that have demonstrated a 50% reduction in their mean Silness-Loë Plaque Index from baseline will be calculated. This will enable the change in the mean plaque index at the individual level to be measured, whilst providing a meaningful primary outcome measure at a population level.

The sample size calculation (see section ‘Sample size {14}’) is based and powered on changes to the POM at 6 months. This takes account of the ‘time-to-effect’ of the intervention on the POM and the pragmatic consideration of the length of stay of eligible residents. However, a follow-up at 12 months will be undertaken to determine whether the effect seen at 6 months can be sustained. This aligns with the eligibility criteria and the estimated length of stay that the residents have in a care home [43].

#### Secondary outcomes

- Clinical outcomes (assessed at baseline, 6 months and 12 months) will include the number of new carious lesions (coronal and root caries) and episodes of bleeding on probing. This will be undertaken on the same index teeth (according to Ramfjord) to reduce the burden on participating residents [44];
- Health−related quality of life (HRQoL) will be assessed at baseline, 6 months and 12 months using the EuroQol five dimensions questionnaire (EQ−5D5L) [45]. The EQ−5D5L is an established HRQoL outcome and is also relevant for a cost−utility analysis;
- Oral health−related quality of life (OHRQoL) will be assessed at baseline, 6 months and 12 months using the Oral Impacts on Daily Performances (OIDP) [46]. This will assess the impacts of oral conditions on daily life. It is a widely used OHRQoL outcome also applied in care homes in the UK;
- Oral symptoms, episodes of pain and episodes of unscheduled care; and
- The number of onward referrals to dentists, when the treatment needed extends beyond the DTs’ Scope of Practice [33].

#### Cost-effectiveness analysis

A parallel cost-effectiveness analysis from an NHS perspective will be undertaken and will examine potential long-term costs and benefits of the intervention. A cost-effectiveness model will be produced.

### Participant timeline {13}

**Fig. 2:**
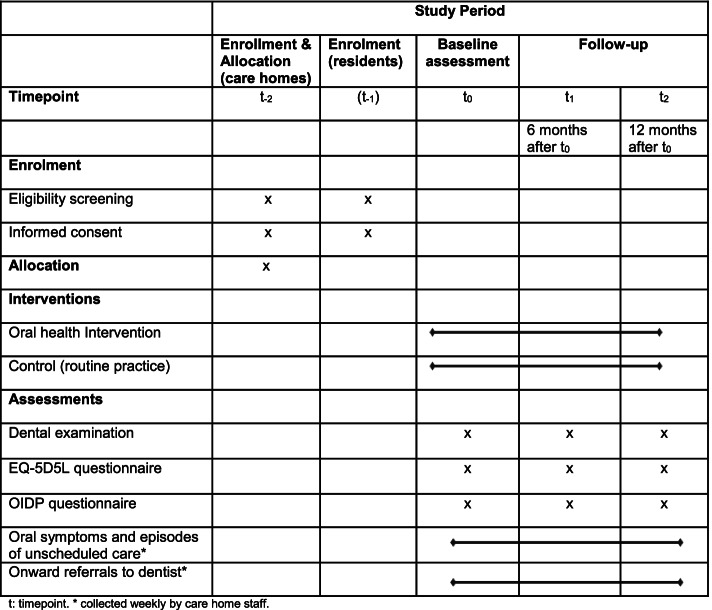
Time schedule of enrolment, interventions, assessments and visits for participants

Figure 2 provides an overview of the study timeline. Care homes will be informed about the study and screened for eligibility (*t*_−2_). Eligible care homes who are interested in taking part will be recruited, then randomly allocated to the intervention or control arm (*t*_−2_). Residents within the recruited care homes will be initially screened by the care home manager. The local study team will then confirm eligibility, and from the residents that have been chosen, select a random sample of eligible residents for the trial (*t*_−1_). Eligible residents or their Personal Consultee will then be asked to provide informed written consent (*t*_−1_).

The care homes allocated to the intervention arm will be asked to implement the oral health intervention during the study period (12 months, *t*_0_ to *t*_2_), and the care homes allocated to the control arm will be asked to continue with routine practice during the same 12-month period. Assessments (intervention and control group) will be undertaken at baseline (*t*_0_), 6 months after baseline (*t*_1_) and 12 months after baseline (*t*_2_).

### Sample size {14}

The primary outcome will be based on a binary outcome using a superiority design, with a successful case demonstrating a 50% reduction in their mean Silness-Löe Plaque Index [42] over a 6-month period, i.e. the relative proportion of the residents whose Silness-Löe Plaque Index have been halved as a result of the intervention will be compared across the two arms. This concurs with two relevant studies, which tested the implementation of an oral hygiene guideline in a comparable population. Khanagar et al. [47] demonstrated a 50% reduction in the mean Silness-Löe Plaque Index (3.17 to 1.57), and the mean reduction amongst residents found by van der Putten et al. [48] was from 2.36 to 1.58. Assuming the proportion of the control group that will halve their mean Silness-Löe Plaque Index over 6 months is 0.10 (given the halo effect of participating in a trial), a sample of 280 (20 care homes per arm with five completers per care home plus attrition at 28%) will provide 90% power to detect a 0.19 difference in proportions between the arms at a 5% significance level (i.e. 0.29 of intervention group will see a 50% reduction in their mean Silness-Löe Plaque Index). This incorporates an intraclass correlation coefficient of 0.05. The measurements taken at 12 months will determine whether this effect (the primary end point being 6 months) can be sustained for a further 6 months.

It is likely that care homes will be able to recruit a variable number of participants due to their relative size and differing populations. Therefore, a coefficient of variation has been included in the sample size to accommodate this. Taking the approach of Campbell and Walters [49] and assuming that recruitment will vary between 5 and 15 participants within a home the sample proposed will accommodate this and still retain 90% power.

### Recruitment {15}

Recruitment will be a two-stage process. The first stage will be the recruitment of the care homes. The study will be conducted in 40 care homes (with expected 50% recruitment rate, 80 homes will be approached). Eligible care homes will be informed about the study through the standard communication routes (letter/email/phone call or in person) and in collaboration with Care Forum Wales [50], ENRICH-Cymru [51] and ENRICH in London [52]. The research team will also use the footprint of participating CDSs across Wales, Northern Ireland, Central and North London and the North of England. If eligible care homes want further information, a member of the local study team will arrange a visit in person to provide the care home managers with an information sheet, further discuss the study and answer any questions about participation. At least 48 h after discussing the study, eligible care homes will be contacted by the researcher to confirm whether they would like to take part or not.

The second stage will be the recruitment of eligible residents in participating care homes. The aim is to recruit approximately seven residents per home resulting in an estimated sample size of 280 residents. The care home managers will be responsible for initially screening the residents to determine who meets the eligibility criteria. All staff involved in the recruitment of participants will be trained on the eligibility criteria prior to site activation. A short presentation about the study will be provided to potentially eligible residents and/or their Personal Consultees. Those interested in participating will be given a PIS and an ICF. The local study team will attend the care home at least 48 h after discussing the study so that potential participants can ask questions about the study and express their interest in taking part. Before any trial-specific procedures are performed, the participant will need to sign and date the ICF.

## Assignment of interventions: allocation

### Sequence generation {16a}

Eligible care homes will be randomised (via the North Wales Organisation for Randomised Trials in Health [NWORTH]) based on a 1:1 ratio (20 intervention and 20 control). Stratification of care homes will be based on care home site (England, Northern Ireland, Wales) and care home size (based on number of available beds within the care home: small 1–10 beds; medium 11–49 beds; large 50 +). NWORTH’s dynamic adaptive algorithm [53] will be used to balance these factors a priori, and these factors will be adjusted for post hoc as part of the analysis.

### Concealment mechanism {16b}

Local study team members will allocate the care homes using a dynamic adaptive randomisation algorithm [53].

### Implementation {16c}

Since this is a cluster RCT, residents will be allocated to the treatment that have been assigned to the care home. An independent NWORTH member of staff will allocate the care homes and a member of the local study team will enrol residents on to the study.

## Assignment of interventions: blinding

### Who will be blinded {17a}

It is not possible to blind the care homes, the individual residents in the trial or outcome assessors, but the trial statistician will remain blind throughout the duration of the study, until the blinded analysis detailed in the statistical analysis plan has been conducted and reported to the study team.

### Procedure for unblinding if needed {17b}

The statistician will be unblinded to allocation after the statistical analysis plan has been conducted and reported to the study team.

## Data collection and management

### Plans for assessment and collection of outcomes {18a}

All study participants (intervention and control arms) will undergo a dental examination to collect clinical data and will complete pre-printed validated questionnaires and a symptom checklist to collect person-centred outcomes. The following assessments will take place at the care home for each recruited resident:

#### Clinical outcomes

Clinical outcomes include the degree of dental plaque present (POM) assessed by Silness-Loë Plaque Index [42]), number of new carious lesions (coronal and root caries) and episodes of bleeding on probing on six index teeth. The dental examination will be undertaken at baseline, 6 months and 12 months by trained dental examiners from the CDS at each study site (CDS organisations are large enough to prevent contamination between measurements of clinical indices and the delivery of the intervention). The dental examination will be undertaken on six index teeth (according to Ramfjord) to reduce the burden on participating residents [44].

#### Person-centred outcomes

- HRQoL will be assessed through the EQ−5D−5L questionnaire [45]. This questionnaire will be administered by a trained member of the local study team to all participants at baseline, 6 months and 12 months;
- OHRQoL will be assessed using the OIDP questionnaire [46]. This questionnaire will be administered by a trained member of the local study team to all participants at baseline, 6 months and 12 months;
- Oral symptoms and episodes of unscheduled care. This refers to the number of reported episodes of dental pain, sepsis, discomfort and urgent dental care appointments. Care home staff using a checklist diary log will collect this information weekly; and
- The number of onward referrals to dentists, when the treatment needed extends beyond the DTs’ Scope of Practice [33], will be collected weekly by care home staff, and by a member of the local study team at baseline, 6 months and 12 months.

#### Other information

On entry into the study, the local study team will complete a Six-item Cognitive Impairment Test (6-CIT) [30] with each resident in order to assess their level of cognitive function. In order to be as inclusive as possible, all residents that are able to provide consent will be included in the study, but a record will be kept of their 6-CIT scores: 0–7 = normal cognitive function; 8–9 = mild cognitive impairment; > 10 = severe cognitive impairment. The impact of this change to the eligibility criteria is reflected in the increase of our modelled attrition rate (28%). A sensitivity analysis will be undertaken at the analysis stage to determine its effect on the POM. A log will be kept of residents who decide not to participate and the number of residents who were unable to provide consent.

#### Cost-effectiveness data

Costs fall under a number of headings and will be gathered under these. Programme delivery costs (DN and DT) in terms of (i) staff time; (ii) travel costs; (iii) consumables (such as toothpaste) and (iv) production of training materials and websites will be collected using a combination of staff diaries and programme records. Staff time will be monetised using estimates of salary costs from published sources; travel time using costs per mile travelled and consumables based on programme acquisition costs. Production of training materials and development of the website will be treated as a fixed cost and based on programme records. Other dental costs will be based on a review of patient records with respect to dental care beyond preventive activities provided by DN and DT or other dental practitioners. This will be monetised using the Statement of Dental Remuneration for Northern Ireland, which provides greater granularity in terms of the identification of fee for service [54].

#### COVID-19

Due to the Coronavirus (COVID-19), remote contact with the study team will be privileged over face-to-face meetings (including trial visits), where possible, to ensure that both participants and staff are protected. Face-to-face trial visits that need to be performed will only be undertaken where care homes have been COVID-free for at least 14 days. Care homes (on behalf of the staff and residents) and researchers will be required to complete a COVID-19 screening tool prior to face-to-face visits to ensure risk is minimised. Visits will then be booked. During the visit, researchers will wear a mask and ensure social distancing and handwashing/hand sanitisation are performed, in line with local rules and national regulations. Before conducting any trial visits at the care home, the researchers will provide their details and a declaration of their own COVID status (no symptoms or contact with known COVID cases for 14 days). The provision of the details of the researchers will enable ‘track and trace’ to be performed, should this be necessary. Due to the sensitive patient population, every precaution will be taken to minimise the risk of infection, with decontamination taking place prior to entering and leaving the resident and the care home.

The number of confirmed cases of COVID19 within the care homes will be recorded at baseline, 6 months and 12 months to assess any impact the research may have had on the home during the study.

### Plans to promote participant retention and complete follow-up {18b}

The research team will have regular contact with the recruited care homes throughout the study period. Care homes will be kept informed of the study’s progress by the study team and via a quarterly email.

### Data management {19}

Information regarding how the data is to be collected, stored, and transferred will be included in the study-specific Data Management Plan. The study will be managed in accordance with General Data Protection Regulations, Good Clinical Practice (GCP) and relevant NWORTH Standard Operating Procedures (SOPs). Where data are stored locally at local sites, the study will adhere to the SOPs of the respective University.

The person-centred measures (questionnaires) will be collected on pre-printed case report forms (CRFs) for each resident. The symptoms checklist completed by care home staff on a weekly basis will be filed and locked in the care home manager’s office, ahead of collection by the local study team. Data from the paper CRFs will be transcribed onto a web-based CRF that will not include the participant’s name or other information that could identify them. The CRF will be considered the source data and should be consistent and verifiable with the information recorded on the study database.

All data will be stored securely on password-protected PCs/laptops and any paper records stored in locked drawers/filling cabinets in secure buildings. All participant personal information will be coded and anonymised at source. Participants will be allocated a unique study number, which will be used in any documentation associated with the study. Participants’ names will not appear on any documentation associated with the study apart from the Consent Forms, which will be kept separate in locked filing cabinets within the resident’s care home. Only members of the research team will have access to the data.

### Confidentiality {27}

Individual participant medical information obtained as a result of this study is considered confidential, and disclosure to third parties is prohibited with the exceptions noted below:

- CRFs will be labelled with a unique trial identification number.
- Medical information may be given to the participant’s medical team and all appropriate medical personnel responsible for the participant’s welfare.

NWORTH will preserve the confidentiality of participants taking part in the study, and the Sponsor is registered as a Data Controller with the Information Commissioners Office.

### Plans for collection, laboratory evaluation and storage of biological specimens for genetic or molecular analysis in this trial/future use {33}

Not applicable as no biological specimens will be collected as part of this trial.

## Statistical methods

### Statistical methods for primary and secondary outcomes {20a}

Primary analysis will be considered at the 6-month end point using a multi-level logistic regression model to assess the differences between the two treatment groups and will accommodate the within care home-level clustering. Analysis will be conducted on an intention to treat basis, and if required, a per-protocol analysis will be conducted as sensitivity analysis. Stratification variables (site and care home size) will be incorporated into the model. All treatment effect estimates will be presented with 95% confidence intervals. Analysis of secondary outcomes will follow the same analysis model as the primary analysis where possible. Binary outcomes will be analysed using multi-level logistic regression and continuous outcomes with a multi-level mixed effects model. Exploratory analysis will be conducted to establish the effect of cognitive impairment on the outcome.

The independent committees will have the opportunity to comment on this plan. If any deviations from the planned statistical analysis are required, these will be fully documented and justified in the final analysis report.

A nested internal pilot will be conducted across all three geographical areas over 3 months using stop/go criteria, assessing the accumulated data using ACCEPT criteria [55]:

1. Recruitment rate of eligible participants (Green > / = 50% + ; Amber 40–49%; Red <39%);
2. Number who remain engaged with the study and that we expect to follow-up at 6 months (Green > / = 72% + ; Amber 61–71%; Red <60%);
3. Fidelity rate from the participating clinicians (Green > / = 80%; Amber 70–80%; Red <69%);
4. Data completion rate for the POM (Green > / = 80%; Amber 70–80%; Red <69%);
5. Confirmation that cost data is available and that appropriate cost information can be collected from the setting to inform the health economic evaluation;
6. Confirmation of the social acceptability of the intervention for residents, formal carers and care home managers; and
7. No adverse events (see adverse events below) that are considered by the Data Monitoring Committee to highlight an unacceptable risk to enrolled participants.

In this manner, the internal pilot will test the trial procedures and check that we can recruit and retain participants. Equally, it will ensure that any adaptations made to the study will still be valid for the main analysis.

#### Cost-effectiveness analysis

Costs will be aggregated for the individual residents based on their consumption of resources under each heading and in total. Relevant costs will be differentiated between those required to ‘treat’ an individual and those required to set up the study. Total treatment costs will be related to outcomes in a series of cost-effectiveness ratios, for oral cleanliness, pain, oral and general health-related quality of life. As analyses are confined to 6 months in the base-case, no discount rate will be applied. In order to quantify the uncertainty associated with the incremental cost-effectiveness ratios (ICERs), a stochastic analysis will be undertaken, with the results presented as a series of cost-effectiveness acceptability curves (CEACs). The CEACs will show the probability of the intervention being cost-effective compared to usual care for a range of maximum monetary values that decision-makers may be willing to pay for the outcome concerned.

Sensitivity analyses will explore variations in unit costs and in the value set used to estimate generic health-related quality of life using a series of deterministic analyses where ICERs will be recalculated. The analysis of costs will be used to identify key drivers of cost and the potential to reduce data collection in future studies. This information will be examined collectively, discussed and used as the basis for recommendations on future data collection and whether and/or which outcomes focus further cost-effectiveness analyses.

### Interim analyses {21b}

There are no interim analyses planned for this trial. Harm suffered by participants from trial participation is not expected; therefore, this trial has no formal stopping guidelines. However, the Date Monitoring Committee will regularly review any adverse events.

### Methods for additional analyses (e.g. subgroup analyses) {20b}

On entry into the study, residents will be asked to complete a 6-CIT questionnaire in order to assess their level of cognitive function. A sensitivity analysis will be undertaken at the analysis stage to determine its effect on the POM.

The number of confirmed cases of COVID19 within the care homes will be recorded at baseline, 6 months and 12 months to assess any impact this may have had on the care home during the study.

### Methods in analysis to handle protocol non-adherence and any statistical methods to handle missing data {20c}

Analysis will be conducted on an intention to treat basis, and if required, a per-protocol analysis will be conducted as a sensitivity analysis. The aim is to minimise the amount of missing data, supported by the limited additional visits required by the participants. However, there is an expectation that some missing data will occur; predictors of missingness will be investigated and will be considered for inclusion in the models. Multiple imputation will be employed to address missing outcomes where appropriate. Test modelling and missing data assumptions via sensitivity analyses will be undertaken.

### Plans to give access to the full protocol, participant-level data and statistical code {31c}

The Statistical Analysis Plan, data and code can be shared upon reasonable justified request.

## Oversight and monitoring

### Composition of the coordinating centre and trial steering committee {5d}

The study will be sponsored by Bangor University and the governance and management of the study will be undertaken by NWORTH on behalf of the Sponsor.

A Trials Management Group (TMG) will oversee the day-to-day running of the study and be composed of research team members and will meet frequently during setup and subsequently on an agreed periodic basis once the trial is open to recruitment.

Trial-specific training requirements will be addressed throughout the study period and regularly reviewed. Working alongside NWORTH’s Quality Assurance Officer, the local study team will co-ordinate oversight of monitoring, documentation and all aspects of quality management and regulatory issues. NWORTH’s Senior Trials Manager will provide advice to the management team on all aspects of the running of the study.

The project’s Trial Steering Committee (TSC) will oversee the running of the trial on behalf of the Sponsor and funder and will have overall responsibility for the continuation or termination of the trial. It will ensure that the trial is conducted in accordance with the principles of Good Clinical Practice and the relevant regulations, and to provide advice on all aspects of the study. The TSC will consist of a range of national and international experts on different aspects of the project, and Patient and Public Involvement (PPI) representatives. The TSC will meet every 6 months.

### Composition of the data monitoring committee, its role and reporting structure {21a}

The project’s Data Monitoring Committee (DMC) will monitor the safety of the study and provide advice on any relevant changes that are required to the conduct of the study via recommendations to the TSC. It consists of three independent members that collectively have expertise on Gerodontology and statistics. DMC will meet every 6 months.

### Adverse event reporting and harms {22}

The adverse events (AEs) reporting period for this study begins as soon as the participant consents to be in the study and ends 1 month after their final data collection ends.

Serious adverse events (SAEs) will be recorded in a running log at each care home. This will include death and hospitalisation due to any cause. Participants’ medical notes will be reviewed for any hospitalisation. Due to the nature of the study population, SAEs are expected. Care home staff will keep a running log of SAEs and this will be regularly collected by the research team and overseen by the Principal Investigators.

SAEs that are related to taking part in the study and are unexpected will be sent onto the Sponsor, the Research Ethics Committee, DMC and the TSC within the required timelines. Should any SAE be associated with the intervention, the local study team will notify the Chief Investigator immediately, who will actively investigate the event. Other adverse events, although unlikely in a health promotion intervention, will be noted in the same log as the SAEs and a monthly report will be compiled. The occurrence of AEs during the trial will be monitored by the DMC and TSC.

### Frequency and plans for auditing trial conduct {23}

A monitoring plan, based on a risk assessment, will be prepared prior to participant recruitment detailing the monitoring strategy for the trial. The plan will include procedures for day-to-day centralised monitoring, process for setting the 6- and 12-month follow-up assessments conducted by DCPs, the requirements for source data verification, Investigator Site File audit, and for identification of protocol deviations and serious breaches of protocol and/or GCP.

### Plans for communicating important protocol amendments to relevant parties (e.g. trial participants, ethical committees) {25}

Any modifications to the protocol will be communicated to all relevant parties including the funder, the sponsor, the ethics committee and other relevant authorities. The trial registry entry will be updated, and all research sites will receive a revised copy to store in their Investigator site file.

### Dissemination plans {31a}

Multiple routes will be taken to the dissemination of the study. The results of the study will be disseminated to the scientific community through conference presentations and peer-reviewed publications. The research team have formal links with dental commissioners, national and international policymakers and regulators such as the Regulation and Quality Improvement authority (RQIA) [56], practising dentists and dental care professionals. A participatory workshop will be held at the end of the study, bringing together these key stakeholder groups along with the participating care homes. The workshop will provide a direct line to disseminate the findings of the study and help tailor a communication plan to reflect the knowledge, interests and concerns of the different stakeholders.

Members of the research team are key figures in several International and European organisations, including the European College of Gerodontology, the International Association of Dental Research, Geriatric Oral Research Group, the Council of European Chief Dental Officers, the Platform for Better Oral Health in Europe and the British Society of Gerodontology. These links provide a direct line for dissemination of the research findings. Strong links with the Adult Oral Health Oversight Group within the National Dental Public Health team in England and the Centre for Policy on Ageing [57] that promotes the interests of older people through research, policy analysis and knowledge transfer, will also be utilised.

Furthermore, the research team have strong links with ENRICH-Cymru in Wales [51] (e.g. VOICE) and Care Forum Wales [50], which represents over 450 care homes, nursing homes and other independent health and social care providers across Wales. The research team will also utilise existing relationships that they have developed with several other organisations in Wales, including the Centre for Ageing and Dementia [58] and the largest third sector organisation in Wales: Age Cymru [59]. In Northern Ireland, collaborative links have been formed with Age Sector Platform which represents a strong unified voice for older people in Northern Ireland [60]. It is the charity responsible for the Northern Ireland Pensioners Parliament. Age Sector Platform has a membership of individuals and older people’s groups across Northern Ireland, representing approximately 200,000 people. These networks will complement BELONG [61] and PARC-Bangor [62], to ensure strong PPI representation. Informal dissemination networks will be made by these PPI groups, who will also link to the Patient and Client Council [63]. This will ensure dissemination of information directly to dependent older people and their carers/relatives and care home managers/staff. Our PPI co-applicants will also ensure a strong patient-facing dissemination strategy. Patient-facing materials will be developed (and also produced in audio-format).

## Discussion

The health needs of the population are changing. Many older adults are now retaining their dentition for longer [64], and this presents significant challenges to dental services. In many countries, the provision of dental services currently adopts a ‘one-size-fits-all’, where the dentist is the main caregiver [18]. DCPs offer an alternative to using dentists to meet the future challenges in dental public health. DCPs have the potential to increase the efficiency and effectiveness of service provision, which ultimately can help to release resources to improve access to oral health care. The feasibility and effectiveness of using DCPs has been tested in primary care [21, 23, 24], but little has been undertaken in a care home environment where dental service provision is often poor with little emphasis on prevention. Evidence on how best to successfully implement and sustain DCP interventions within the care home environments is warranted.

This protocol paper describes the design of a cluster RCT, based on a superiority design, that will be used to determine the effectiveness of using DCPs (DTs and DNs) in the care home environment. The study will help strengthen the evidence base regarding the use of DCPs as an alternative to dentists in the care home environment. Furthermore, the parallel process evaluation will provide valuable information on how the intervention could successfully be embedded in standard practice.

## Trial status

The study protocol is version 3 (18th March 2021). The trial is currently in the pre-recruitment phase. Recruitment of care homes will hopefully commence 1 March 2022. Trial completion will be expected by July 2024.

## Supplementary Information

**Additional file 1.** Consent form (trial residents). Consent form (care-homes).

## Data Availability

At the end of the study NWORTH will release a data pack containing the raw data extracted from MACRO database, the analysis data sets and any agreed syntax. The Statistical Analysis Plan, data and code can be shared publicly upon reasonable justified request.

## Abbreviations

AE: Adverse event
CDS: Community dental service
CEACs: Cost-effectiveness acceptability curves
COVID19: Coronavirus disease 2019
CRF: Case Report Form
DCPs: Dental Care Professionals
DMC: Data Monitoring Committee
DNs: Dental nurses
DTs: Dental therapists
EQ-5D5L: EuroQol five dimensions questionnaire
GCP: Good Clinical Practice (GCP)
HRQoL: Health-related quality of life
ICERs: Incremental cost-effectiveness ratios
ICF: Informed consent form
NHS: National Health Service
NICE: National Institute of Clinical Excellence
NIHR: National Institute for Health Research
NWORTH: North Wales Organisation for Randomised Trials in Health
OHRQoL: Oral health-related quality of life
OIDP: Oral Impacts on Daily Performances
PHE: Public Health England
PIS: Participant Information Sheet
POM: Primary Outcome Measure
PPI: Patient and Public Involvement
RCT: Randomised controlled trial
SAE: Serious adverse event
SOP: Standard Operating Procedures
TOPIC: Improving the oral health of older people in care homes
TMG: Trial Management Group
TSC: Trial Steering Committee
TIDieR: Template for Intervention Description and Replication
6-CIT: 6 Item Cognitive Impairment Tool
