# Supplementary material for: uSing rolE-substitutioN In care homes to improve ORal health (SENIOR): a study protocol
Source: Trials. 2022 Aug 18;23:679. doi: 10.1186/s13063-022-06487-3 (PMC9386206; doi:10.1186/s13063-022-06487-3)
Supplement: Supplementary file 1 — Additional file 1. Consent form (trial residents). Consent form (care-homes). [file 13063_2022_6487_MOESM1_ESM.zip › 6-CF_trial_residents_V1R1.docx]

**CONSENT FORM (TRIAL RESIDENTS)**

**Study title:** uSing rolE-substitutioN In care-homes to improve oRal health (SENIOR)

**Name of Researcher:** Professor Paul Brocklehurst

**IRAS ID:** 297182

**Study Number:** NIHR_128773

**Centre Number:**

**Participant Identification Number for this trial:**

Please initial each box.

1. I confirm that I have read the information sheet dated.................... (version............) for the above study. I have had the opportunity to

consider the information, ask questions and have had these answered satisfactorily;

1. I understand that my participation is voluntary and that I am free to

withdraw at any time without giving any reason, without my medical care

or legal rights being affected;

1. I understand that the information collected about me will be used to

support other research in the future and may be shared anonymously

with other researchers;

1. I understand that I will not be identifiable in any data published in relation

to this project;

1. I understand that relevant data collected during the study, may be

looked at by individuals from Bangor University, Queen’s University

Belfast, University College London, Cardiff University, University of

Sheffield, regulatory authorities or from the NHS, where it is relevant

to my taking part in this research; and

1. I agree to take part in the above study, inclusive of all the procedures

mentioned in the Information Sheet. I understand that my personal

information will be used for the purposes explained to me. I understand

that according to data protection legislation, ‘public task’ will be the lawful

basis for processing.

1. I understand that at the start of the study, I will be a given a Six Item

Cognitive Impairment Test and that the results of this test will be passed

onto my care-home manager under the duty of care obligations.

**Name of participant:**

Date:

Signature:

**Name of person taking consent:**

Date:

Signature:

**When completed and signed: 1 copy for participant; 1 copy for researchers site file; 1 (original) to be kept in participant notes**
