# Supplementary material for: uSing rolE-substitutioN In care homes to improve ORal health (SENIOR): a study protocol
Source: Trials. 2022 Aug 18;23:679. doi: 10.1186/s13063-022-06487-3 (PMC9386206; doi:10.1186/s13063-022-06487-3)
Supplement: Supplementary file 1 — Additional file 1. Consent form (trial residents). Consent form (care-homes). [file 13063_2022_6487_MOESM1_ESM.zip › CF_trial_care-homes_V1R1.docx]

**CONSENT FORM (CARE-HOMES)**

**Study title:** uSing rolE-substitutioN In care-homes to improve oRal health (SENIOR)

**Name of Researcher:** Professor Paul Brocklehurst

**IRAS ID:** 297182

**Study Number:** NIHR_128773

**Centre Number:**

**Participant Identification Number for this trial:**

Please initial each box.

1. I confirm that I have read the information sheet dated.................... (version............) for the above study. I have had the opportunity to

consider the information, ask questions and have had these answered satisfactorily;

1. I understand that the participation of our care-homes is voluntary and

that we are free to withdraw at any time without giving any reason,

without our legal rights being affected;

1. I understand that the information collected about our care-homes will

be used to support other research in the future and may be shared

anonymously with other researchers;

1. I understand that we will not be identifiable in any data published in

relation to this project;

1. I understand that relevant data collected during the study, may be

looked at by individuals from Bangor University, Queen’s University

Belfast, University College London, Cardiff University, University of

Sheffield, regulatory authorities or from the NHS, where it is relevant

to my taking part in this research; and

1. I agree for our care-home to take part in the above study, inclusive

of all the procedures mentioned in the Patient Information Sheet.

I understand that information will be used for the purposes explained

to us. I understand that according to data protection legislation, ‘public

task’ will be the lawful basis for processing.

**Name of care-home manager:**

Date:

Signature:

**Name of person taking consent:**

Date:

Signature:

**When completed and signed: 1 (original) for care-home and 1 copy for researchers site file.**
